# Toward a Phenotype-Driven Continuum Model in Trigger Finger: Proposing a Sonographic Framework for Personalized Management

**DOI:** 10.3390/life16020289

**Published:** 2026-02-08

**Authors:** Sang-Hyun Kim, Jihyo Hwang, Yonghyun Yoon, Jaeik Choi, Gyungseog Ko, Hyeongjik Kim, Dongyeun Sung, Rowook Park, Jaehyun Shim, Jonghyeok Lee, Seungbeom Kim, Youngmo Kim, King Hei Stanley Lam

**Affiliations:** 1 College of Korean Medicine, Woosuk University, 443, Samnye-ro, Samnye-eup, Wanju_gun, Jeonju 55338, Republic of Korea; amalang@catholic.ac.kr; 2 Department of Orthopaedic Surgery, Gangnam Sacred Heart Hospital, Hallym University College of Medicine, 1 Singil-ro, Yeongdeungpo-gu, Seoul 07441, Republic of Korea; 3 Incheon Terminal Orthopedic Surgery Clinic, Inha-ro 489beon-gil, Namdong-gu, Incheon 21574, Republic of Korea; 4 International Academy of Regenerative Medicine, Inha-ro 489beon-gil, Namdong-gu, Incheon 21574, Republic of Korea; jic121@hanmail.net (J.C.); kgs8368@hanmail.net (G.K.); congkalu23@naver.com (H.K.); 9854069@gmail.com (D.S.); prwook@naver.com (R.P.); jhyunshim@gmail.com (J.S.); perfectceive@gmail.com (J.L.); stplayer@naver.com (S.K.); rmkimym@gmail.com (Y.K.); 5 International Association of Musculoskeletal Medicine, Kowloon, Hong Kong, China; 6 MSKUS, 1035 E. Vista Way #128, Vista, CA 92084, USA; 7 Dr. Choi’s Rehabilitation Clinic, Daelim Building 303, Kojandong, Ansansi, Kyengido, Seoul 15476, Republic of Korea; 8 Ahyeon Orthopedic Clinic, B270 Sinchon-ro, Mapo-gu, Seoul 04116, Republic of Korea; 9 Himchannamu Neurosurgery Clinic, 439, Hakjeong-ro, Buk-gu, Daegu 41423, Republic of Korea; 10 Department of Rehabilitation Medicine, Sae Yonsei Rehabilitation Clinic, Seoul 03186, Republic of Korea; 11 Department of Neurosurgery, Chungdammadi Neurosurgery Clinic, Seoul 03186, Republic of Korea; 12 Bareun Neurosurgery Clinic, 39, Daenong-ro, Heungdeok-gu, Cheongju-si 28402, Republic of Korea; 13 Miso Pain Clinic, 1569, Bongyeong-ro, Yeongtong-gu, Suwon-si 16703, Republic of Korea; 14 Hongje Oneul Teun Teun Clinic, 442, Tongil-ro, Seodaemun-gu, Seoul 03629, Republic of Korea; 15 Faculty of Medicine, The University of Hong Kong, Hong Kong, China; 16 Faculty of Medicine, The Chinese University of Hong Kong, Hong Kong, China; 17 The Department of Clinical Research, The International Association of Musculoskeletal Medicine, Hong Kong, China; 18 The Department of Clinical Research, The Hong Kong Institute of Musculoskeletal Medicine, Hong Kong, China

**Keywords:** trigger finger, stenosing tenosynovitis, ultrasonography, sonographic phenotyping, A1 pulley, ultrasound-guided interventions

## Abstract

Background: The traditional A1-centric paradigm for trigger finger (TF) management does not fully capture heterogeneous pathology spanning isolated pulley stenosis, tendon degeneration, and impaired tendon–sheath gliding. Methods: A comprehensive literature synthesis (2010–2025) integrating anatomy, biomechanics, and ultrasound-guided interventions was performed to develop a testable, phenotype-driven framework. Results: A continuum model is proposed emphasizing (i) origin-to-insertion assessment of the flexor apparatus, (ii) pragmatic ultrasound phenotyping into pulley-dominant, tendon-dominant, and mixed patterns, and (iii) a stepwise, phenotype-matched management pathway incorporating conservative care, ultrasound-guided injection, selected adjuncts (e.g., hydrodissection, prolotherapy, ESWT) for tendon-dominant or mixed presentations, and percutaneous or open release when an A1 bottleneck is confirmed. Conclusions: This framework is presented as a hypothesis to guide standardized reporting, reliability testing, and phenotype-stratified comparative trials, rather than as a validated clinical guideline. This article proposes a novel, phenotype-driven clinical framework to address this limitation. Contemporary evidence is integrated to construct a model emphasizing (i) a whole-length, origin-to-insertion assessment of the flexor apparatus, (ii) sonographic phenotyping into pulley-dominant, tendon-dominant, and mixed patterns, and (iii) a stepwise treatment algorithm integrating conservative care, ultrasound-guided injections, ultrasound-guided percutaneous release, and selected adjunctive approaches such as hydrodissection (HD), prolotherapy (Prolo), and extracorporeal shockwave therapy (ESWT). While evidence supports individual modalities, the framework’s primary innovation lies in matching interventions to phenotype. This sonographic phenotyping system is presented not as a validated tool, but as a testable hypothesis designed to guide future validation studies. The proposed framework establishes research priorities, including standardized criteria, reliability testing, and comparative effectiveness research for phenotype-stratified management.

## 1. Introduction

Trigger finger (TF), clinically termed stenosing tenosynovitis, is a frequent hand disorder characterized by painful catching, clicking, or locking of a digit during flexion–extension [1]. It contributes substantially to functional limitation in daily activities and work, and it is frequently encountered in general orthopedic and primary practice [2,3]. Although TF can affect any digit, the thumb and ring finger are commonly involved [4], and the condition is associated with systemic comorbidities such as diabetes mellitus, inflammatory arthropathies, and thyroid disease [5]. Critically, recent genetic evidence establishes a causal link with carpal tunnel syndrome, underscoring potential shared pathomechanical pathways within the flexor apparatus [6]. Standard clinical grading systems describe a continuum from intermittent triggering to fixed locking, reflecting a progressive disturbance of tendon excursion within the fibro-osseous tunnel [7].

Historically, TF has been predominantly explained as a focal stenosis at the A1 pulley level, where pulley thickening and secondary flexor tendon changes impede smooth gliding. This “A1-centric” paradigm has supported conventional treatment strategies centered on corticosteroid injection (CSI) into the flexor sheath and surgical A1 pulley release [8]. While these approaches are effective for many patients, this model does not fully account for the well-documented clinical heterogeneity. This includes persistent or recurrent symptoms after injection or release, atypical pain localization, multi-level mechanical symptoms, and cases in which sonographic or intraoperative findings suggest dominant tendon pathology rather than isolated pulley stenosis [9]. In addition, the term “tenosynovitis” implies inflammatory predominance, whereas many histopathologic and imaging observations are more consistent with degenerative or mixed tendinopathic changes within a mechanically constrained environment [10,11].

Consequently, a pathoanatomical continuum model has gained traction. This concept is particularly salient when TF coexists with proximal flexor apparatus dysfunction. The flexor digitorum superficialis (FDS) and profundus (FDP) are not isolated digital structures; they belong to a continuous muscle–tendon unit originating from the forearm—functionally linked to the common flexor origin at the medial epicondyle—and inserting distally into the phalanges. Along this origin-to-insertion pathway, mechanical overload, enthesis-related pain, or myotendinous dysfunction can alter tendon tension, excursion patterns, and load-sharing during gripping [12,13]. Thus, digital triggering may represent the distal manifestation of a broader flexor unit disorder in a subset of patients. This perspective helps to explain heterogeneous pain distributions, incomplete symptom resolution after A1-directed treatment, and recurrent mechanical symptoms despite technically adequate pulley-focused interventions [5].

High-resolution ultrasound has emerged as a pivotal modality for both diagnostic clarification and procedural guidance in TF [7,14,15]. Beyond confirming pulley and tendon thickening, ultrasound allows dynamic assessment during finger motion to reproduce triggering phenomena, identify the level of mechanical conflict, and evaluate the flexor apparatus along its length [16]. This capability enables sonographic phenotyping— categorizing cases into pulley-dominant, tendon-dominant, or mixed patterns—which can be used to align interventions with the presumed primary pain generator and mechanical bottleneck. Moreover, ultrasound guidance facilitates minimally invasive strategies including sheath injections, percutaneous A1 release, and hydrodissection (HD) of the tendon–sheath or interfascial planes, potentially improving accuracy while limiting iatrogenic injury when performed with appropriate technique [17,18,19].

Therefore, the purpose of this article is to move beyond a traditional narrative review. Contemporary evidence is synthesized to propose and justify a novel, testable clinical framework for the management of TF. This framework is anchored in an origin-to-insertion view of the flexor apparatus and is designed to address the limitations of the A1-centric paradigm. It articulates (i) a continuum-based anatomical and biomechanical model from the common flexor origin to distal insertions, (ii) a pragmatic system for ultrasound phenotyping to identify pulley-dominant, tendon-dominant, and mixed pathology drivers, and (iii) a hypothesis-driven, stepwise treatment algorithm integrating conservative care, ultrasound-guided biologic and mechanical interventions, and percutaneous or open release. This phenotype system is presented as a construct requiring future validation, with the aim of guiding phenotype-stratified research and advancing personalized clinical practice.

## 2. Anatomy (With Cadaver Correlation)

**Figure 1:**
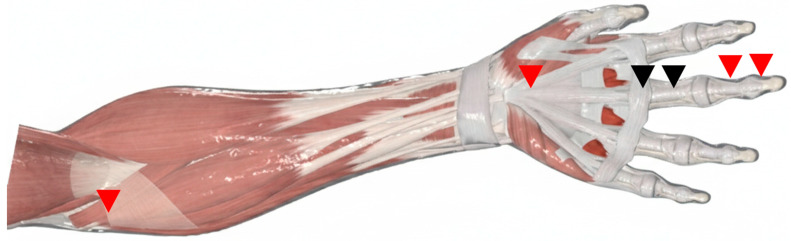
Schematic illustration of the flexor tendon–pulley–sheath continuum in trigger finger. The diagram highlights potential pathological sites beyond the classic A1 pulley. Conventional primary targets (e.g., the A1 pulley) are indicated by black arrowheads. Additional potential sites of mechanical conflict relevant to tendon-dominant or mixed phenotypes (e.g., the A0 pulley, segmental tendon pathology, and proximal flexor origin) are indicated by distinct (red) arrowheads in the figure.

The flexor tendon–pulley system of the hand is a biomechanically efficient apparatus designed to maximize tendon excursion while maintaining stable, low-friction digital flexion [20]. In TF, symptoms emerge when this system loses its smooth gliding behavior due to structural or functional mismatch within the flexor tendon–pulley–sheath continuum. Accordingly, a precise anatomical understanding provides the foundation for ultrasound-based phenotyping and supports the rationale for extending treatment considerations beyond an A1-only paradigm (Figure 1).

### 2.1. The Pulley System: Functional Segmentation

The digital pulley system comprises five annular pulleys (A1–A5) and three cruciform pulleys (C1–C3), forming a retinacular tunnel that keeps the flexor tendons closely opposed to the phalanges. This configuration prevents bowstringing and converts muscular pull into efficient flexion across the metacarpophalangeal (MCP) and interphalangeal joints. Functionally, the pulleys act as segmental constraints that shape tendon course and contact mechanics during motion—features that become clinically relevant when pulley thickening, tendon enlargement, or adhesions alter the tendon–pulley interface.

**The A0 pulley (palmar aponeurotic pulley).** Proximal to the A1 pulley, an additional constraining structure—often referred to as the A0 pulley or palmar aponeurotic pulley—has been described at the level of the palmar aponeurosis. As a proximal component of the flexor retinacular system, it may contribute to tendon constraint at the entrance of the digital apparatus and has been proposed as a potential contributor to triggering symptoms in selected cases. Recent cadaveric evidence confirms that constriction at the A0 pulley alone is sufficient to induce triggering, with a reported clinical incidence as a primary source in 31% to 47% of patients. Recognition of the A0 pulley supports a broader, origin-to-insertion interpretation of the flexor unit and underscores that mechanical conflict may arise proximal to the classic A1 level [21,22].

**Figure 2:**
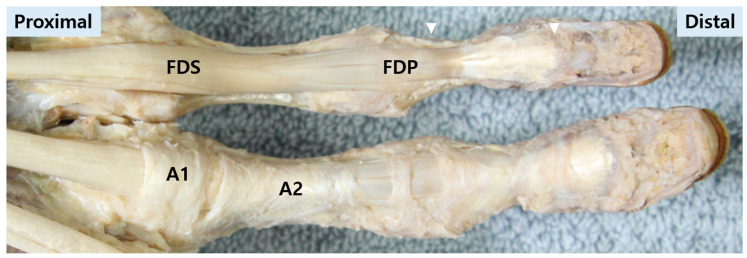
Cadaveric dissection demonstrating the flexor tendon system and digital pulleys. A comparative view of the fourth (superior) and third (inferior) fingers is shown. In the fourth finger, the pulley system has been removed to expose the underlying flexor digitorum superficialis (FDS) and flexor digitorum profundus (FDP) tendons. In the third finger, the intact pulley system (A1, A2 pulleys) is preserved. White triangles indicate the distal insertion sites of the FDS and FDP tendons on the middle and distal phalanges, respectively.

**The A1 pulley.** As the primary pulley of interest in TF, the A1 pulley is a robust annular structure located at the volar MCP level (Figure 2). It functions as a key constraint at the entrance to the digital sheath. Its proximal edge is consistently located at the digital palmar crease, with a mean length varying from 8.1 mm in the little finger to 10.7 mm in the middle finger, providing critical landmarks for percutaneous procedures [23]. Pathologic thickening or reduced compliance at this level can increase friction and precipitate the characteristic catching or locking during tendon excursion [24,25].

**The A2 and A4 pulleys.** The A2 and A4 pulleys are located at the proximal and middle phalanges, respectively, and are critical for maintaining normal flexion biomechanics (Figure 2) [26]. These are considered the major “biomechanical” pulleys, with the A4 pulley being particularly crucial for work and excursion efficiency; their preservation is paramount during surgical intervention [20]. Although these pulleys are typically preserved in standard TF surgery, altered tendon routing or stress redistribution after A1 release may increase distal contact forces, providing a plausible anatomical basis for persistent symptoms in selected cases and supporting a continuum-based interpretation of TF [27].

### 2.2. The Flexor Tendons: A Journey from Origin to Insertion

Pathology in TF is not necessarily confined to the A1 pulley. The flexor tendons themselves may exhibit segmental degeneration, nodularity, or insertional changes that contribute to mechanical symptoms and pain. Therefore, an “origin-to-insertion” anatomical perspective can be clinically informative—analogous to tendon-origin–focused paradigms used in other enthesopathy-driven conditions.

**Flexor digitorum superficialis (FDS).**

**Figure 3:**
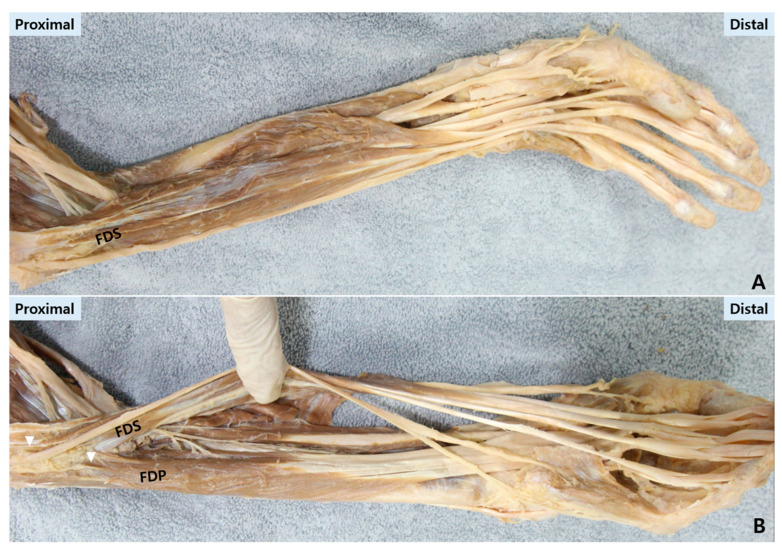
Cadaveric demonstration of the flexor digitorum superficialis (FDS) and profundus (FDP) tendons along their course. (**A**) The FDS tendon is traced from its muscular origin in the proximal forearm to its bifurcation and insertion on the middle phalanx. (**B**) The FDP tendon is shown originating from the proximal ulna and interosseous membrane. Removal of the pulley system demonstrates the resulting bowstringing of the tendon, a key biomechanical consequence. White triangles mark the proximal origin sites of the FDP and FDS.

**Origin (proximal).** The FDS arises from the common flexor origin and adjacent forearm structures, giving rise to four tendons that travel through the carpal tunnel into the digit (Figure 3A).

**Insertion (distal).** Each FDS tendon bifurcates at the level of the proximal phalanx (Camper’s chiasm) into two slips that course around the FDP tendon and insert on the middle phalanx [28]. This bifurcation creates an anatomically complex region that may be susceptible to focal thickening, frictional interaction, and stiffness when tendinopathic changes develop. On ultrasound, the FDS is typically visualized superficial to the FDP at the level of the A1 pulley and is the tendon most commonly affected by the rubbing and locking phenomenon in TF [29].

**Flexor digitorum profundus (FDP).**

**Origin (proximal).** The FDP originates from the proximal ulna and interosseous membrane, traveling through the carpal tunnel deep to the FDS tendons (Figure 3B).

**Insertion (distal).** The FDP passes through the FDS split and inserts on the distal phalanx [30]. Segmental tendon enlargement, surface irregularity, or distal insertional pathology may contribute to impaired gliding and global digital stiffness, especially when combined with pulley constraint [31,32]. Ultrasound allows for the distinct identification of both the FDS and FDP tendons along their course, which is essential for dynamic assessment and identifying tendon-dominant pathology [29].

### 2.3. The Synovial Sheath and the “Low-Friction Plane”

The flexor tendons are enclosed within a double-walled synovial sheath that supports low-friction motion. Within this sheath, a functional “low-friction plane” facilitates tendon excursion; disruption of this plane by synovial hypertrophy, fibrosis, or adhesions can increase friction and amplify triggering phenomena, even when focal pulley thickening is modest [33]. This anatomical concept also provides a mechanistic rationale for ultrasound-guided strategies aimed at restoring tendon mobility by separating adherent layers and reducing frictional resistance. Sonographically, the normal synovial sheath appears as a thin, hypoechoic rim surrounding the hyperechoic tendon fibrils; its thickening or effusion is a key diagnostic finding [29,34].

### 2.4. Clinical Implication of the Continuum Model

A continuum-based anatomical model reframes TF as a disorder of constrained tendon gliding rather than an isolated A1 lesion. This is supported by the histopathological finding of fibrocartilaginous metaplasia and increased Type III collagen at the tendon–pulley interface, indicative of a degenerative, mechanically driven process [20]. This perspective motivates comprehensive assessment of the flexor apparatus and supports individualized, target-based interventions when symptoms persist despite conventional A1-focused management—paralleling how treatment in other enthesis-related disorders is directed toward the most relevant segment (e.g., tendon origin) rather than a single anatomic bottleneck.

## 3. Biomechanics and Pathophysiology

### 3.1. The Pulley System as a Biomechanical Constraint

Digital flexion relies on a retinacular pulley system that keeps the flexor tendons closely opposed to the phalanges, maximizing mechanical efficiency and preventing bowstringing [35]. The annular pulleys act as segmental constraints that define tendon routing and contact mechanics; small changes in pulley integrity or tendon contour can therefore produce disproportionate changes in friction, shear, and excursion behavior. The interaction can be modeled as a cable (tendon) moving around a fixed rod (pulley), where frictional forces are a critical determinant of gliding efficiency [36]. In this setting, trigger finger represents a failure of smooth tendon gliding within a constrained tunnel rather than a purely static narrowing.

### 3.2. Pathomechanics of Trigger Finger: Mismatch, Friction, and a Dynamic Threshold Event

From a mechanical perspective, TF arises when the effective tendon volume becomes mismatched with the compliance and caliber of the pulley tunnel and sheath environment. This mismatch may reflect pulley thickening or stiffening, tendon enlargement or nodularity, or loss of the low friction gliding plane due to fibrosis or adhesions [35]. This process aligns with a continuum model of tendinopathy, where the primary pathology is a failed adaptive response of the extracellular matrix to mechanical load, rather than a classic inflammatory “tenosynovitis”. Histopathological findings of fibrocartilaginous metaplasia at the tendon–pulley interface support this degenerative, mechanically driven process. As contact pressure increases at the tendon–pulley interface, friction and shear rise until tendon motion becomes intermittently obstructed. Biomechanical studies measuring the gliding of the flexor digitorum profundus tendon through the A2 pulley have quantified the coefficient of friction in a relevant gliding model to be approximately 0.016 ± 0.005 [36]. This low-friction state is essential for normal function and is disrupted in TF. Clinically, this manifests as a dynamic “threshold” event: the tendon advances under tension and then abruptly snaps past the point of maximal resistance, with symptoms modulated by motion direction and load. Importantly, sonographic evidence indicates that flexor tendon thickening occurs progressively and is observable even before the onset of active clinical triggering in digits other than the thumb, underscoring the continuum of structural change [37].

### 3.3. Why “A1-Only” Is Not Universally Benign: Biomechanics-Based Mechanisms of Suboptimal Outcomes

The A1 pulley is often the most accessible and frequently implicated bottleneck; however, an A1-only paradigm can be biomechanically incomplete for two reasons. First, frictional resistance in TF can be distributed along the tendon–sheath continuum, such that reducing the A1 constraint may not normalize global tendon excursion when tendon-dominant degeneration or adhesion-related gliding restriction remains. Second, altering a focal retinacular constraint can change load sharing across the pulley system, shifting contact pressures and shear to adjacent segments.

A clinically important implication is that release performed beyond the intended target—whether through inadvertent extension of division or anatomical variation—may compromise pulley integrity and alter tendon routing [38]. When the A1 pulley is contiguous with or closely approximates the A2 pulley, release that functionally involves the A2 component can produce unfavorable biomechanics, including increased bowstringing tendency, altered tendon moment arms, and secondary changes in flexion mechanics. These biomechanical changes can lead to metacarpophalangeal subluxation, bowstring phenomenon, and ulnar deviation, which in turn affect active range of motion [35].

### 3.4. Anatomical–Biomechanical Variation: The A1–A2 Interface as a Risk Zone

Although the A1 pulley is the conventional target in TF surgery, the functional boundary between A1 and A2 may not always be sharply demarcated in practice. When the two pulleys are anatomically close or partially confluent, the procedural “safety margin” for release narrows [39]. In such scenarios, the risk is not simply incomplete symptom relief but also unintended biomechanical consequences if the A2 pulley’s restraining function is compromised. This is particularly relevant for minimally invasive or percutaneous approaches, where direct visualization is limited and where small deviations in level or depth can change which pulley fibers are divided.

### 3.5. Implications for This Review

These pulley biomechanics considerations help explain why outcomes and complications after A1-focused procedures may vary across patients and techniques and why indiscriminate escalation to release is not always the most coherent strategy. Accordingly, this article synthesizes anatomical, biomechanical, and ultrasound-guided evidence to support mechanism-based decision-making—prioritizing careful target definition, ultrasound-informed localization of the dominant bottleneck, and stepwise escalation from conservative care and injection-based strategies to release procedures when clearly indicated.

## 4. Materials and Methods

This work was conceived as a comprehensive review and conceptual synthesis, aimed at constructing a novel clinical framework rather than providing a traditional narrative summary. The objective was to integrate contemporary evidence into a coherent, phenotype-driven model for ultrasound-guided management of trigger finger (TF).

The framework development followed a multi-stage, iterative process: (1) comprehensive evidence identification, (2) thematic synthesis aligned with a flexor apparatus continuum model, and (3) formal proposition of sonographic phenotypes and a matched treatment algorithm.

### 4.1. Evidence Identification and Selection

A systematic search strategy was designed to capture the breadth of relevant literature. PubMed/MEDLINE, and Web of Science Core collection were searched for English-language articles published between January 2010 and December 2025. Search terms included combinations of the following keywords and Medical Subject Headings (MeSH) where applicable: “trigger finger disorder”, “tenosynovitis”, “flexor tendon”, “tendinopathy”, “ultrasonography”, “A1 pulley”, “percutaneous release”, “hydrodissection”, “prolotherapy”, and “extracorporeal shockwave therapy”. Eligible sources included randomized trials, cohort and case–control studies, case reports and case series, cross-sectional imaging studies, anatomical/cadaveric studies, biomechanical studies, systematic reviews, and high-quality narrative reviews that addressed anatomy, biomechanics, diagnostic ultrasound, and/or interventions relevant to trigger finger. Conference abstracts without full text, non-peer reviewed sources, non-English articles, and Publications unrelated to trigger finger pathophysiology, ultrasound evaluation, or treatment were excluded. Additional relevant studies were identified by manual screening of reference lists of retrieved reviews and seminal articles.

### 4.2. Synthesis and Framework Development

Findings from the identified literature were synthesized qualitatively. The synthesis was guided by the overarching hypothesis of a pathoanatomical continuum along the flexor tendon–pulley–sheath unit. Evidence was organized and analyzed to address three core pillars of the proposed framework:

1. The anatomical and biomechanical rationale for an origin-to-insertion perspective.
2. The role of ultrasound in diagnosing the site of conflict and distinguishing patterns of pathology.
3. The evidence base for various minimally invasive interventions, from injections to percutaneous release.

From this synthesis, the pragmatic sonographic phenotypes (pulley-dominant, tendon-dominant, and mixed patterns) were derived as a central, organizing construct. These phenotypes are presented explicitly as a testable classification system intended to guide future hypothesis-driven research and validation studies. Finally, the synthesized evidence was translated into the proposed stepwise, phenotype-matched treatment algorithm.

## 5. Clinical Diagnosis and Ultrasound Phenotyping

### 5.1. Clinical Diagnosis: History, Examination, and Practical Grading

TF is primarily a clinical diagnosis characterized by painful clicking, catching, or locking during active finger motion. Patients often describe symptoms that are worse in the morning or after repetitive gripping tasks, with localized tenderness near the volar metacarpophalangeal (MCP) region in typical cases. Palpation may reveal a tender nodule or thickening along the flexor tendon pathway, and symptoms can often be reproduced by asking the patient to flex and extend the involved digit under light resistance [1].

In some patients, TF-like symptoms coexist with proximal flexor unit pain or dysfunction, including medial elbow pain consistent with involvement of the common flexor origin [40]. Clinically, this may present as pain provoked by gripping, wrist flexion, or pronation, with digital symptoms that appear disproportionate to focal A1 tenderness. Recognizing a proximal driver is important because a purely A1-directed strategy may not fully address symptom persistence in this subgroup.

Clinical severity can be described using pragmatic grading systems such as the Quinnell classification (from intermittent triggering to fixed locking). In routine practice, documentation of (i) triggering frequency, (ii) locking episodes requiring passive release, (iii) range-of-motion limitation, and (iv) functional impact is sufficient to guide initial management and follow-up comparisons [41].

**Differential diagnosis**. Key alternatives include arthritis, flexor tendon rupture or partial tear, volar plate injury, Dupuytren-related contracture, and tenosynovitis in inflammatory arthropathies. Red flags warranting broader evaluation include disproportionate swelling, persistent rest pain, rapidly progressive stiffness, systemic inflammatory features, or atypical pain distribution inconsistent with a flexor mechanism [4].

### 5.2. Why Ultrasound Matters: From Confirmation to Mechanism-Based Targeting

Ultrasound (US) complements clinical assessment by (i) confirming pulley and tendon abnormalities, (ii) identifying the level of mechanical conflict, (iii) enabling dynamic reproduction of triggering phenomena, and (iv) supporting a phenotype-based framework for target selection. Rather than treating TF as a uniform A1 lesion, US facilitates assessment along the flexor tendon–pulley–sheath continuum, helping to distinguish pulley-dominant from tendon-dominant or mixed patterns. It serves as the critical imaging tool for operationalizing the proposed continuum model.

### 5.3. Ultrasound Scanning Protocol

A practical US protocol should include both static morphology and dynamic mechanics.

**Patient positioning and probe orientation.** The hand rests comfortably supinated. A high-frequency linear transducer is used. The evaluation begins at the volar MCP level and then follows the flexor tendons distally and proximally as needed, using both longitudinal and transverse views.

**Core static assessment.**

1. A1 pulley region (volar MCP level):
  ○ Visual assessment of A1 pulley thickening (>0.4–0.5 mm is often considered pathological)
  ○ Assessment of flexor tendon caliber immediately deep to the pulley.
2. Flexor tendons (FDS/FDP):
  ○ Tendon thickening, focal irregularity, and echotexture changes suggestive of tendinopathy [42].
  ○ Segmental assessment along the proximal phalanx and into distal segments when symptoms or tenderness are distal.
3. Tendon sheath and peritendinous tissues:
  ○ Sheath thickening, fluid, or fibrotic-appearing interfaces (when present).
4. Power Doppler (optional but informative):
  ○ Hyperemia at the pulley–tendon interface, sheath, or focal tendon regions can support active tissue response, while acknowledging that Doppler findings are variable and not mandatory for diagnosis.

While the core ultrasound assessment focuses on the digital pulley–tendon–sheath interface, an expanded scan along the flexor unit may be considered when symptoms or examination suggest a proximal driver (e.g., concomitant medial epicondylar tenderness or pain with resisted wrist flexion/pronation). In such cases, evaluating the flexor-pronator origin and proximal myotendinous segments can support an origin-to-insertion phenotyping approach.

**Dynamic assessment (essential).**

Dynamic imaging is performed during slow active flexion–extension. The examiner attempts to reproduce the patient’s symptomatic catching/locking while visualizing the tendon–pulley interface. Additional maneuvers such as gentle resisted flexion or light gripping can be used to increase tendon load and improve reproducibility. The key dynamic observation is whether the triggering event localizes primarily at the A1 level or appears distributed/associated with segmental tendon irregularity and altered excursion along the continuum.

When clinical history or examination suggests a proximal contribution (e.g., grip-load sensitivity or concomitant medial elbow discomfort), the scan may be pragmatically extended along the flexor unit to support an origin-to-insertion interpretation within the tendon-dominant phenotype.

### 5.4. Suggested Ultrasound Measurements (Pragmatic, Not Overly Prescriptive)

Given variability in measurement techniques across studies, this review emphasizes a pragmatic approach. When feasible, the following parameters may be recorded for longitudinal follow-up and phenotype assignment:

- **A1 pulley thickness** (measured at the volar MCP level in a standardized plane).
- **Flexor tendon thickness** (FDS/FDP at or near the A1 level and at the most abnormal-appearing segment).
- **Presence of focal tendon nodularity/irregularity** and its approximate level (MCP/proximal phalanx/middle phalanx/distal segment).
- **Sheath features** (fluid, thickening, or fibrotic interface).
- **Dynamic localization** of the triggering event (A1-focused vs. multi-level/segmental).

These measurements are primarily intended to enhance within-patient tracking and communication, rather than to impose rigid cut-offs.

### 5.5. Sonographic Phenotyping: Minimal Criteria and Clinical Meaning

Based on the anatomical–biomechanical continuum model, TF can be categorized into proposed pragmatic sonographic phenotypes that inform target selection. The following criteria are presented as a testable classification system derived from the integrated evidence in this framework (Table 1).

**Pulley-dominant phenotype.**

**Figure 4:**
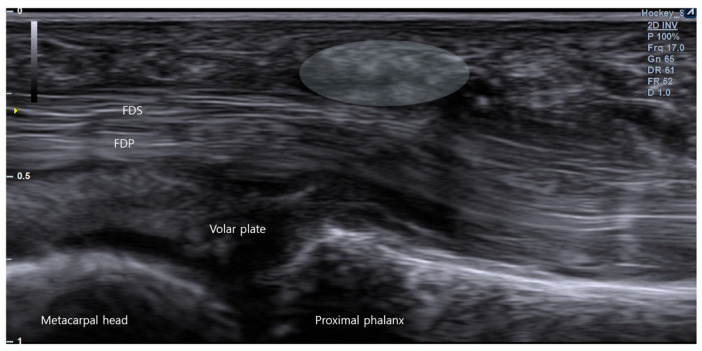
Ultrasonographic demonstration of A1 pulley thickening in trigger finger. A longitudinal sonographic view over the volar aspect of the metacarpophalangeal (MCP) joint. Pathological thickening of the A1 pulley is visualized as a hypoechoic bulge (demarcated by the white shaded area) overlying the flexor digitorum superficialis (FDS) and profundus (FDP) tendons.

**Figure 5:**
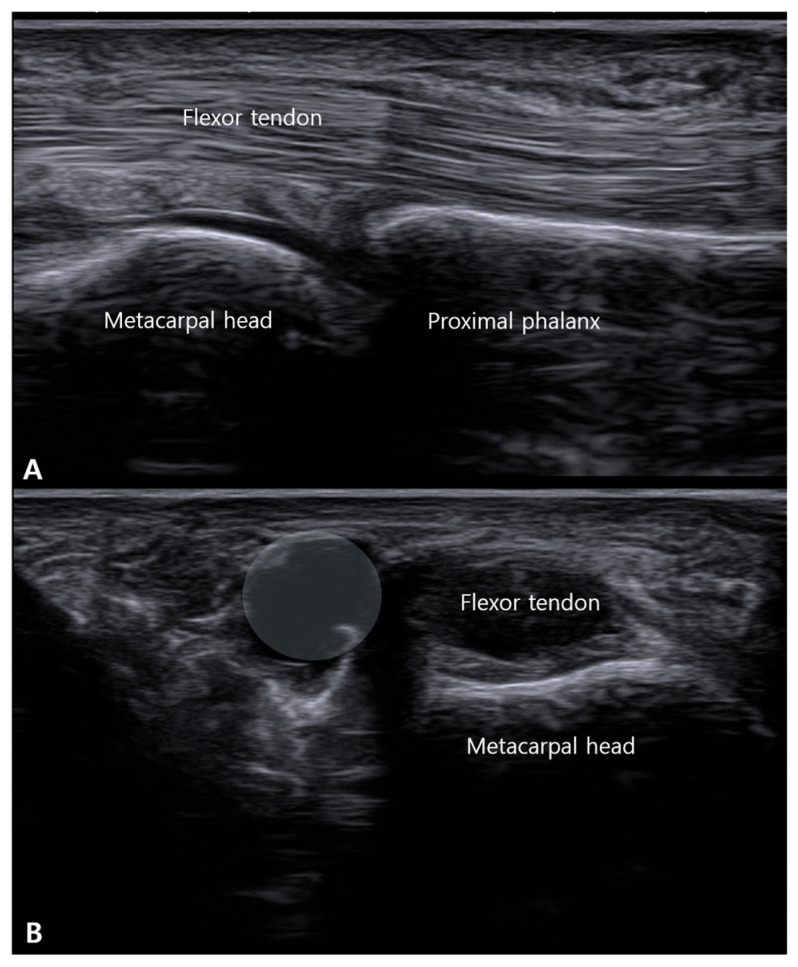
The biplanar ultrasound assessment for detecting an A1 pulley ganglion. (**A**) Longitudinal sonographic view over the A1 pulley region showing no apparent abnormality of the flexor tendons. (**B**) Corresponding short-axis (transverse) view at the same level reveals a well-defined hypoechoic ganglion cyst (whited shaded region) located on the radial aspect of the pulley. This case underscores the necessity of orthogonal imaging planes to avoid missing laterally situated pathology.

**Figure 6:**
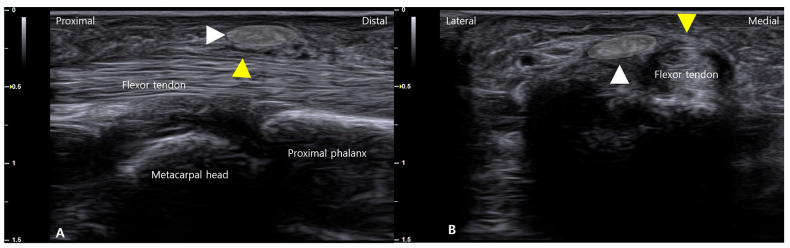
Multimodal ultrasonographic localization of an A1 pulley calcification. (**A**) Long-axis view showing a hyperechoic calcific deposit (white arrowhead within white-shaded area) positioned superficial to the A1 pulley (yellow arrowhead). (**B**) Corresponding short-axis view confirming the calcific focus (white arrowhead within white-shaded area) on the lateral aspect of the A1 pulley (yellow arrowhead). This orthogonal assessment precisely defines the lesion’s spatial relationship to the pulley for sonographic diagnosis and procedural planning.

This phenotype is characterized by a predominant A1-level mechanical constraint with relatively limited tendon abnormality beyond the immediate pulley region. Typical ultrasound findings include clear A1 pulley thickening (Figure 4) with focal impingement and dynamic triggering that localizes primarily to the A1 level, while the tendon contour along the remaining course appears comparatively preserved. Importantly, a pulley-dominant pattern does not imply a uniform or “simple” A1 thickening alone. The pulley complex may harbor additional focal lesions—such as retinacular ganglion change (Figure 5B), calcific foci [43] (Figure 6), or localized pulley irregularity—that can contribute to symptoms or alter procedural targeting. Therefore, ultrasound evaluation should not be confined to a single midline longitudinal view; rather, a focused, pulley-centered survey should include radial and ulnar sweeps and short-axis interrogation around the A1 region to avoid missing lateralized ganglion-like lesions (Figure 5A,B or calcification and to better define the true site of mechanical conflict.

**Tendon-dominant phenotype (origin-to-insertion flexor unit).**

**Figure 7:**
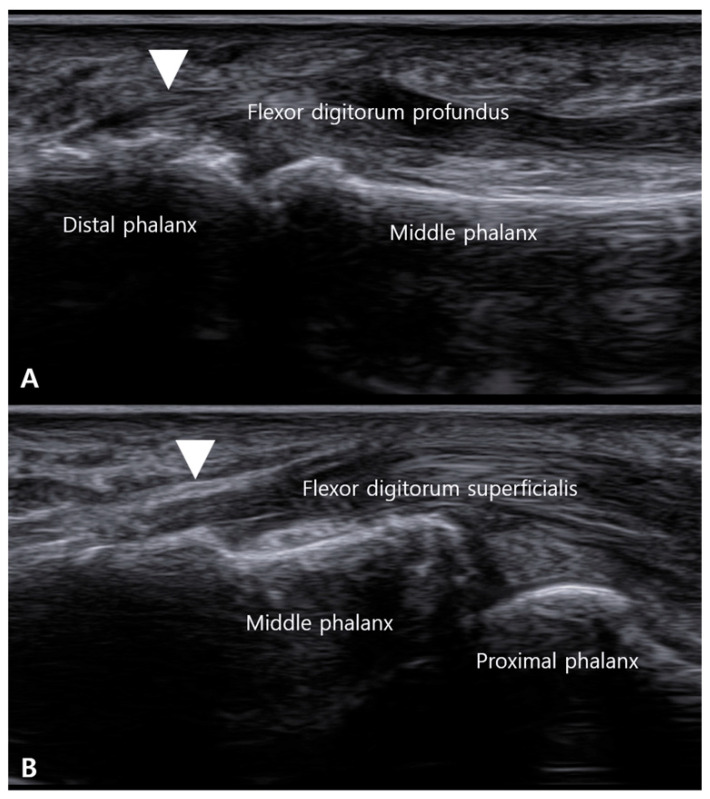
Sonographic evidence of insertional tendinopathy in the tendon-dominant phenotype. Long-axis ultrasound views demonstrate enthesopathic changes at the flexor tendon insertions. (**A**) At the distal phalanx, a palmar bony ridge (white arrowhead) is seen at the insertion of the flexor digitorum profundus (FDP). (**B**) At the middle phalanx, a similar bony ridge (white arrowhead) is present at the insertion of the flexor digitorum superficialis (FDS). These findings represent potential distal drivers of triggering in the tendon-dominant phenotype.

**Figure 8:**
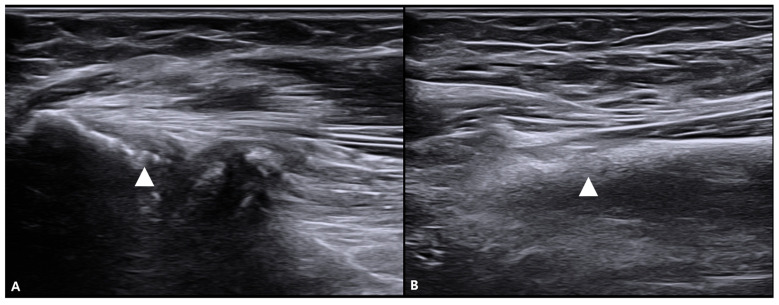
Proximal sonographic findings in the tendon-dominant phenotype. Ultrasound images demonstrate focal calcific/enthesopathic changes at the proximal flexor compartment within the common flexor origin region. (**A**) Calcification (white arrowhead) is visualized at the proximal origin region of the flexor digitorum superficialis (FDS) in the forearm. (**B**) Corresponding calcification (white arrowhead) is seen at the origin region of the flexor digitorum profundus (FDP). These proximal findings exemplify the expanded anatomical scope of assessment in the proposed origin-to-insertion framework; when accompanied by power Doppler activity or other inflammatory features, an inflammatory enthesitis phenotype (e.g., psoriatic arthritis) should be considered in the differential diagnosis.atic arthritis) when accompanied by power Doppler activity or other inflammatory features.

This phenotype reflects a broader flexor apparatus disorder in which the tendon/muscle–tendon unit is the principal driver of symptoms, and digital triggering represents a distal manifestation of impaired gliding or load transfer. It includes segmental tendinopathic changes along the digital tendons (e.g., focal thickening, nodularity, contour irregularity, or insertion-related pathology, which may encompass enthesopathic changes such as insertional irregularity and adjacent bony ridging at the phalanges [44] (Figure 7)) and may also involve proximal dysfunction at the flexor muscle–tendon unit, including the common flexor origin region at the medial epicondyle (Figure 8).

Clinically, patients may report pain distribution or provocation patterns (e.g., grip-related symptoms, wrist flexion/pronation load sensitivity, or concomitant medial elbow discomfort) that appear disproportionate to isolated A1 tenderness. Ultrasound may show tendon-dominant abnormalities along the symptomatic segment(s) with triggering mechanics that are not fully explained by A1 morphology alone.

Because tendon-dominant insertional (enthesopathic) changes may also reflect an inflammatory process, psoriatic arthritis (PsA) and other spondyloarthropathies should be considered when tendon-dominant insertional findings raise concern for an inflammatory “mini-enthesitis” phenotype. On ultrasound, entheseal thickening, enthesophyte formation (new bone at the insertion), and increased power Doppler signal may support active enthesitis rather than purely mechanical overload. When these inflammatory features are present—particularly alongside pulley-related inflammation—clinical correlation (history of psoriasis and inflammatory symptoms) and rheumatologic evaluation may be warranted.

**Mixed phenotype.**

This phenotype is assigned when both a meaningful A1-level constraint and substantial tendon-dominant abnormalities coexist, and the clinical presentation plausibly reflects combined pathology.

### 5.6. Clinical Utility: Linking Phenotype to a Stepwise Strategy

Phenotyping is not intended to replace clinical judgement but to provide a common, mechanism-based language for target selection. As a derived construct of this framework, its utility is hypothetical and must be validated. In pulley-dominant TF, A1-centered strategies (e.g., sheath injection and, when indicated, percutaneous or open A1 release) are often biomechanically coherent. In tendon-dominant or mixed phenotypes, additional attention to the broader tendon–sheath continuum may be warranted, including strategies aimed at restoring the gliding plane and addressing segmental tendon pathology or adhesions. This phenotype-driven approach also provides a testable framework for future phenotype-stratified comparative trials.

## 6. Management: A Phenotype-Driven, Stepwise Approach

### 6.1. Rationale and Overarching Principles

TF management can be framed as progressive reduction in friction and restoration of tendon excursion within the flexor tendon–pulley–sheath continuum. While A1 pulley release can be highly effective in pulley-dominant disease, an indiscriminate A1-only strategy may be insufficient when the dominant driver is distributed along the flexor unit (tendon-dominant) or when combined pathology exists (mixed). Ultrasound phenotyping is proposed as a pragmatic method to localize the principal mechanical bottleneck and to match interventions to target(s) in a stepwise manner, prioritizing lower-risk options before escalation to release procedures. Importantly, this phenotype-matched algorithm is presented as a theoretical, hypothesis-driven proposal; at present, there are no randomized trials demonstrating superiority of phenotype-matched care over standard approaches, and validation is needed before it can be considered a clinical guideline.

**Phenotype linkage**

- **Pulley-dominant:** A1-level constraint is the primary bottleneck → A1-centered strategies are typically coherent.
- **Tendon-dominant (origin-to-insertion flexor unit):** tendon/muscle–tendon unit pathology or gliding-plane impairment is the main driver → strategies restoring excursion and addressing distributed friction may be required; this phenotype may include proximal flexor unit contribution (e.g., common flexor origin region).
- **Mixed:** both components matter → combined strategy and rehabilitation emphasis.

### 6.2. First-Line: Conservative Care

Conservative management aims to reduce tendon load and repetitive irritation while maintaining pain-limited motion. Typical measures include activity modification, splinting for 6–10 weeks (often limiting MCP flexion), and manual therapy focusing on edema control and controlled range-of-motion [41,45,46]. Conservative care is most appropriate for mild or intermittent triggering and can be used as an initial step in all phenotypes, particularly when symptoms are early or functional impact is limited [47,48,49].

**Practical escalation rule**: If symptoms remain functionally limiting or locking persists despite an adequate trial of conservative care, procedural options may be considered based on phenotype and patient context.

### 6.3. Second Line: CSI, Preferably Ultrasound-Guided

CSI remains a widely used minimally invasive intervention aimed at reducing local tissue response and friction at the tendon–pulley interface. Ultrasound guidance facilitates accurate placement (e.g., intrasheath or peripulley plane) and supports documentation of the intended target. In pulley-dominant presentations, CSI can be a highly rational next step before release [50]. In tendon-dominant or mixed patterns, CSI may provide partial relief but may be insufficient if distributed tendon pathology or gliding-plane restriction is dominant.

**Precautions:** CSI can be associated with a transient pain flare and minor injection-site bleeding/bruising. Local cutaneous effects such as skin hypopigmentation and subcutaneous (fat) tissue atrophy may occur. Patients with diabetes should be counseled about possible short-term hyperglycemia. Infection is rare, but consent and post-procedure instructions should explicitly address this risk, including the possibility of deep-site infection. Very rarely, tendon or pulley rupture has been reported, particularly after repeated corticosteroid injections [51,52].

### 6.4. Third Line (Phenotype-Matched): Restoring the Gliding Plane and Addressing Tendon-Dominant Drivers

When ultrasound suggests a tendon-dominant or mixed phenotype—particularly when symptoms are not fully explained by A1 morphology—interventions that aim to restore the low-friction gliding plane may be considered. Ultrasound-guided HD can be used to separate adherent planes around the tendon–sheath interface or adjacent interfascial layers, with the goal of improving excursion and reducing shear [53]. In addition, selected adjunctive approaches (e.g., prolotherapy (Prolo) or extracorporeal shockwave therapy) may be discussed as phenotype-matched options when clinical features suggest a broader tendinopathic component across the flexor unit (origin-to-insertion concept) [43,44,54,55]. These approaches should be framed as targeted adjuncts rather than universal steps, acknowledging heterogeneity in indications, technique, and current evidence.

**Precautions:** HD or needle-based adjunctive interventions may cause transient soreness, swelling, or bruising. Because technique and target planes vary across reports, procedural details (plane, needle trajectory, injectate) should be explicitly documented to support reproducibility and safety.

### 6.5. Fourth Line: A1 Pulley Release (Ultrasound-Guided Percutaneous vs. Open)

A1 pulley release is typically considered when triggering or locking is frequent, functionally limiting, or refractory to nonoperative measures. Ultrasound-guided percutaneous release has emerged as a minimally invasive alternative to open surgery and may offer practical advantages in selected settings [56,57,58,59]. Open release provides direct visualization and may be preferred when anatomy is uncertain, when atypical pathology is suspected, or after failed prior procedures.

In a phenotype-driven framework, pulley-dominant TF is most biomechanically aligned with A1 release. In tendon-dominant and mixed patterns, A1 release may still be appropriate if dynamic imaging localizes a meaningful A1 bottleneck; however, persistent symptoms after technically adequate release should prompt consideration of remaining tendon-dominant drivers and the need for rehabilitation or additional continuum-targeted management.

**Precautions:** For percutaneous release, incomplete release can lead to persistent triggering or early recurrence; both percutaneous and open approaches may be associated with postoperative pain and stiffness, and open release can involve scar-related symptoms or wound concerns. Residual pain after release may reflect phenotype mismatch or unaddressed continuum pathology rather than procedural failure alone.

### 6.6. Rehabilitation, Follow-Up, and Outcome Documentation

Across phenotypes and interventions, early restoration of pain-limited motion, edema control, and gradual return to load-bearing activities are commonly emphasized to reduce stiffness and functional limitation. Follow-up should document triggering resolution, range of motion, pain distribution (including proximal flexor unit symptoms when relevant), functional recovery, and recurrence.

**Proposed stepwise pathway**

Conservative care → CSI → (HD or Prolo, ESWT in tendon-dominant or mixed cases, or in residual symptoms) → Ultrasound-guided A1 release or Open A1 release (Table 2).

This sequence supports escalation from lower-risk, reversible interventions to definitive release when clinically indicated, while minimizing indiscriminate A1-only procedures.

## 7. Pitfalls and Risk Mitigation

Because adverse events and “treatment failure” in TF are often interpreted inconsistently across studies, a compact, mechanism-based summary can improve clinical translation. Persistent or recurrent symptoms after A1-directed interventions should be differentiated into (i) technical inadequacy (e.g., incomplete release) versus (ii) phenotype mismatch, where tendon-dominant or mixed drivers remain unaddressed. Across modalities, stiffness and residual pain may reflect delayed mobilization, ongoing distributed friction along the flexor tendon–sheath continuum, or proximal flexor unit load-transfer issues within the tendon-dominant spectrum. Therefore, risk mitigation should emphasize (a) clear procedural target definition and documentation (injection plane, HD plane, release confirmation), (b) early pain-limited motion and structured follow-up, and (c) phenotype-aware escalation rather than indiscriminate A1-only management (Table 3).

## 8. Discussion

This article presents a continuum-based interpretation of TF as a disorder characterized by restricted tendon motion within the flexor tendon–pulley–sheath unit. The primary etiology may be pulley-dominant, tendon-dominant along the flexor tendon unit from its origin to its insertion, or mixed. This framework provides a consistent biomechanical explanation for clinical heterogeneity and the failure of A1-centric strategies alone to completely resolve symptoms in some patients. Ultrasound phenotyping provides a practical method for localizing key mechanical bottlenecks and targeting minimally invasive interventions to the most appropriate site, supporting a stepwise treatment pathway from conservative to injection-based treatments, gliding surface restoration approaches in tendon-dominant/mixed presentations, and, if indicated, A1 release (ultrasound-guided percutaneous or open procedures).

However, the accumulated evidence to date remains heterogeneous. Direct comparisons and broad generalizations are difficult because of variability in ultrasound definitions, treatment techniques (e.g., injection sites, HD targets, percutaneous release devices), outcome measures, and follow-up periods across studies. Furthermore, practical phenotyping criteria are not standardized, and studies stratifying and analyzing outcomes by phenotypic ultrasound are rare. Given these limitations, over-reliance on specific techniques should be avoided, and the need for systematic reporting standards should be emphasized.

Diagnostic boundaries are important when expanding the tendon-dominant phenotype to the proximal flexor unit. In this framework, medial epicondylar pain is not proposed as a standalone ‘trigger finger phenotype’ in isolation. Rather, proximal flexor-unit involvement is considered only when (i) digit triggering remains the defining clinical problem, (ii) ultrasound demonstrates tendon-continuum abnormalities (digital and/or proximal flexor-pronator unit), and (iii) symptom provocation suggests load-transfer along the flexor unit (e.g., grip-load sensitivity with concordant digital symptoms). In contrast, isolated medial epicondylitis typically presents with dominant medial elbow pain provoked by resisted wrist flexion/pronation without characteristic digital catching/locking; in such cases it should be classified and managed as a comorbidity rather than a driver of digital triggering. Explicitly distinguishing ‘tendon-dominant TF with proximal flexor-unit features’ from ‘TF with concomitant medial epicondylitis’ improves clinical interpretability and should be tested in future validation studies.

An additional boundary condition is inflammatory disease. Trigger finger may occur in spondyloarthropathy, especially psoriatic arthritis, where the A1 pulley can behave as a functional mini-enthesis. In these patients, ultrasound may show A1 pulley thickening accompanied by power Doppler signal, and distal insertional changes may reflect mini-enthesitis rather than purely degenerative tendinopathy. Accordingly, when clinical context suggests inflammatory arthropathy (e.g., psoriasis, inflammatory back pain, dactylitis, or polyenthesitis), the proposed phenotyping should incorporate an ‘inflammatory pulley-dominant’ consideration and prompt appropriate systemic assessment and comorbidity-aware management.

Future studies should prioritize (i) standardized ultrasound definitions for pulley, tendon, and entheseal findings (including Doppler criteria where relevant), (ii) intra- and inter-rater reliability testing for the proposed phenotypes, and (iii) phenotype-stratified comparative effectiveness research to determine whether phenotype-matched interventions improve outcomes compared with standard A1-centric care. Importantly, the treatment pathway presented here is a theoretical, hypothesis-driven proposal rather than a validated clinical guideline, and it should be interpreted as a framework for future trials and reporting standards rather than an established standard of care.

## 9. Conclusions

Trigger finger is proposed to represent a continuum of constrained flexor tendon gliding disorders rather than a uniform, isolated A1 pulley lesion. A pragmatic ultrasound phenotypic classification (pulley-predominant, tendon-predominant along the origin-to-insertion flexor unit, or mixed) is presented to support mechanism-based communication and to generate testable treatment hypotheses. A stepwise, phenotype-matched pathway is proposed (conservative care → ultrasound-guided CSI → selected glide-surface/tendon-continuum adjuncts such as HD, Prolo, or ESWT in tendon-predominant or mixed cases → ultrasound-guided percutaneous or open A1 release when a clinically relevant A1 bottleneck is confirmed). Inflammatory contexts (e.g., psoriatic arthritis-related mini-enthesitis) should be considered when interpreting pulley and entheseal findings. Overall, the framework is intended to guide standardized reporting and future phenotype-stratified validation studies rather than to define a clinical standard of care.

## Figures and Tables

**Table 1:** Minimal criteria for pragmatic ultrasound phenotyping (proposed).

Domain	Pulley-Dominant	Tendon-Dominant (Origin–Insertion)	Mixed
Primary driver	A1-level constraint	Tendon/muscle–tendon unit abnormality along the flexor continuum	Combined
Typical pain pattern	Volar MCP-centered	Along digit and/or proximal flexor unit; may include grip-load sensitivity or medial elbow discomfort	Variable
Static US emphasis	Prominent A1 thickening; focal impingement	Segmental tendon thickening/nodularity/irregularity (digital) ± proximal unit involvement	Both present
Dynamic US localization	Triggering mainly at A1	Triggering mechanics not fully explained by A1; multi-segment excursion disturbance	Variable

**Table 2:** Proposed (hypothesis-driven) intervention matrix by phenotype. Abbreviations: CSI, corticosteroid injection; HD, hydrodissection; Prolo, prolotherapy; ESWT, extracorporeal shockwave therapy; UGPR, ultrasound-guided percutaneous release.

Step	Intervention	Primary Target	Best Suited Phenotype	Notes
1	Conservative (splint/therapy)	Load reduction, controlled excursion	All	First-line in most cases
2	US guided CSI	Sheath/peripulley modulation	Pulley-dominant > mixed	Recurrence possible
3	HD Prolo ESWT	Gliding plane/tendon unit modulation	Tendon-dominant, mixed	Use as phenotype-matched adjunct
4	UGPR or open A1 release	A1 constraint removal	Pulley-dominant; selected mixed	Consider open A1 release if anatomy uncertain/failed prior

**Table 3:** Common pitfalls, failure modes, and mitigation by intervention. Abbreviations: CSI, corticosteroid injection; HD, hydrodissection; Prolo, prolotherapy; UGPR, ultrasound-guided percutaneous release.

Intervention	Common Adverse Events	Key Pitfall/Failure Mode	Practical Mitigation
CSI (US-guided)	Flare, bruising, skin depigmentation/atrophy; transient hyperglycemia	Recurrence; inappropriate expectation of “definitive” cure	Counsel on recurrence; document injection plane; diabetes counseling and monitoring
HD Prolo	Transient soreness, swelling, ecchymosis	Nonstandard target/technique; residual tendon-dominant driver	Define and document dissected plane; standardized reporting; reassess phenotype if incomplete response
UGPR (A1 release)	Pain, hematoma, stiffness	Incomplete release vs. phenotype mismatch	Continuous visualization; confirm dynamic symptom mechanics post-procedure; structured rehab
Open A1 release	Scar tenderness, wound discomfort, stiffness	Persistent symptoms from non-A1 drivers	Patient selection; early mobilization; reassess for tendon-dominant/mixed drivers if pain persists

## Data Availability

No new datasets were created or analyzed in this study. Data sharing is not applicable to this article. All schematic illustrations and ultrasound/cadaver images are original work created by the authors for this manuscript.
